# Impact of the Welsh Government Flying Start programme on children’s BMI and immunisation uptake

**DOI:** 10.23889/ijpds.v9i5.2783

**Published:** 2024-09-18

**Authors:** Roshan Toby, Daniel Thayer, Ieuan Scanlon, Amy Stuart

**Affiliations:** 1 Swansea University

## Objective

The Cohort Builder package aims to streamline the cohort building process using R, with simplicity and efficiency as the primary focus. Researchers can generate cohorts from TRE (Trusted Research Environment) datasets by specifying simple cohort definitions as YAML (YAML Ain't a Markup Language) parameters using this package, eliminating the need to write complex queries.

## Approach

Cohort Builder offers researchers a user-friendly interface for defining cohort criteria by abstracting away the complexities of database queries using YAML configuration files. The package enables researchers to focus on defining the desired cohort characteristics rather than navigating the database and tables. Users can easily specify inclusion and exclusion criteria, demographic constraints, temporal constraints, and other dataset parameters to create cohorts tailored to their research goals and reproduce the results using the same configuration file.

## Result

Using the Cohort Builder, users may quickly create cohorts from TRE datasets with minimum code. The package facilitates reproducibility, transparency, and increased productivity. Researchers gain from having to rely less on specialised database expertise and having more flexibility when defining and exploring cohorts.

## Conclusion

Cohort Builder offers a straightforward yet effective method for defining cohorts. The package frees researchers from having to worry about intricate technological details in favour of their analytical objectives.

## Implications

Moving forward, Cohort Builder will support creative research projects across a range of disciplines and promote collaboration among researchers by allowing them to share configuration files for their cohorts.

